# Design and Implementation of a Quartz Cilia MEMS Vector Hydrophone

**DOI:** 10.3390/mi17091061

**Published:** 2026-09-06

**Authors:** Ziming Ren, Shijie Yang, Rushan Xie, Zhonggang Zhang, Libo Gao, Chenyang Xue

**Affiliations:** 1 Laoshan Laboratory, Qingdao 266237, China; 2 Marine Engineering Institute, Jimei University, Xiamen 361021, China; 3 School of Intelligent Manufacturing (Pen-Tung Sah Institute of Micro-Nano Science and Technology), Xiamen University, Xiamen 361102, China

**Keywords:** vector hydrophone, MEMS, quartz cilia, high sensitivity

## Abstract

The cilium avoids energy loss due to bending deformation during vibration, thereby efficiently transmitting the acoustic driving force to the cross-beam. Meanwhile, a hollow cylindrical structure is adopted instead of the traditional solid one. While maintaining the same external geometry, this hollow design not only increases the effective acoustic area to enhance the acoustic driving force but also reduces the added mass of the cilium. This material–structure synergistic optimization improves the acoustic-to-mechanical transfer efficiency of the cilium in low-frequency acoustic fields, thus enhancing the device’s acoustic sensitivity in the low-frequency range. Parametric simulations are conducted using the COMSOL Multiphysics 6.3 platform to systematically optimize key geometric parameters such as the outer radius and height of the quartz cilium, and the optimal dimensions are determined. The sensitivity and directivity of the prototype are measured in the 20–1000 Hz frequency range using the standard hydrophone comparison method. Experimental results show that the quartz cilium vector hydrophone achieves a sensitivity of –179.1 dB (1 kHz, 0 dB = 1 V/μPa) at 1000 Hz. The frequency response curve exhibits good flatness in the low-frequency range and agrees well with the theoretical 6 dB per octave increase characteristic of pressure gradient sensors. Directivity tests reveal a typical figure-8 pattern at both 315 Hz and 630 Hz, with null depths exceeding 30 dB, demonstrating excellent vector detection capability. This study provides an effective approach for improving the low-frequency sensitivity of MEMS (Micro-Electromechanical System) vector hydrophones, and the designed device meets the requirements for underwater target detection with promising prospects for engineering applications.

## 1. Introduction

Oceans cover approximately 71% of the Earth’s surface and harbor abundant biological, mineral, and energy resources. With global advances in science and technology and the growing capacity for ocean exploitation, competition among nations for marine resource exploration and maritime rights has become increasingly intense. However, the marine water body, as a unique physical medium, poses inherent challenges to information transmission. Electromagnetic and optical waves experience severe attenuation in seawater, with extremely limited effective propagation distances, making them inadequate for medium-to long-range underwater information transmission. In contrast, acoustic waves, which are mechanical waves, experience low propagation loss and can travel long distances in water, thus serving as the primary carrier for underwater information transmission [1,2,3].

The overall performance of a sonar system depends, to a large extent, on the performance of its core sensor—the hydrophone. Conventional scalar hydrophones can measure only sound pressure and must rely on large-scale arrays to achieve target localization. In contrast, vector hydrophones are capable of synchronously acquiring both sound pressure and particle velocity vector information at a single measurement point, thereby enabling single-point directional measurement. They offer significant advantages in system miniaturization, resistance to isotropic noise interference, and target identification. MEMS technology offers key advantages, including miniaturization, high integration density, and batch manufacturability. Integrating MEMS technology with the vector hydrophone design concept can endow underwater detection systems with low power consumption, favorable concealment, and ease of array deployment and has thus become an important development direction for next-generation underwater acoustic sensing technology [4,5,6,7].

Since the 1950s, when American researchers developed the first particle velocity vector hydrophone SV-1 vector hydrophone technology has undergone a continuous evolution from conventional piezoceramic designs to MEMS-integrated solutions [8]. Since then, vector hydrophones have evolved along three main technological routes: piezoelectric, fiber-optic, and biomimetic types. Piezoelectric vector hydrophones achieve acoustoelectric conversion through the direct piezoelectric effect of piezoelectric materials. In recent years, significant progress has been made in three-dimensional simultaneous measurement and deep-sea pressure-resistant packaging, yet their low-frequency sensitivity remains constrained by the matching relationship between the material’s piezoelectric constants and the structural capacitance. Fiber-optic vector hydrophones offer advantages such as strong immunity to electromagnetic interference and ease of multiplexing, and performance improvements have been realized through integrated structural design and push-pull operating modes. However, the high system complexity and cost limit their large-scale engineering applications. In this context, biomimetic cilium-based MEMS vector hydrophones have attracted widespread attention due to their simple structure, low power consumption, and ease of array integration. [9,10]. In 2007, Xue’s group at North University of China developed a biomimetic cilia-type vector hydrophone based on the MEMS piezoresistive effect and bionic principles [11]. Its sensitive structure consists of a cross-beam cantilever and biomimetic cilia, achieving a sensitivity of −197.7 dB and an operating frequency band covering 20–1000 Hz. Subsequently, this group successively proposed various cilium configurations, including “jellyfish,” “otolith-shaped,” “cross-ring-shaped,” “hollow mushroom-shaped,” and “cap-shaped” designs, aiming to enhance sensitivity by enlarging the acoustic receiving area or optimizing mass distribution [12,13,14,15,16,17]. However, these designs still suffer from a trade-off between improving low-frequency sensitivity and maintaining the operating bandwidth, which poses challenges in meeting the growing demand for detecting weak low-frequency signals.

To address the above issues, this paper proposes a novel sensitive structure for MEMS vector hydrophones based on a quartz hollow cilium. This structure exploits the high stiffness and low internal friction of quartz to effectively reduce mechanical energy dissipation during cilium vibration. Meanwhile, the hollow cylindrical configuration significantly increases the acoustic interaction area and reduces the added mass while maintaining a constant outer diameter, thereby synergistically overcoming the sensitivity–bandwidth trade-off from both material and structural perspectives. This paper systematically describes the operating principle and mechanical model of the device, conducts parametric simulation and optimization, completes device fabrication and packaging integration, and verifies its performance using a standing-wave-tube calibration system.

## 2. Sensor Principle

### 2.1. Bionic Principle

**Figure 1:**
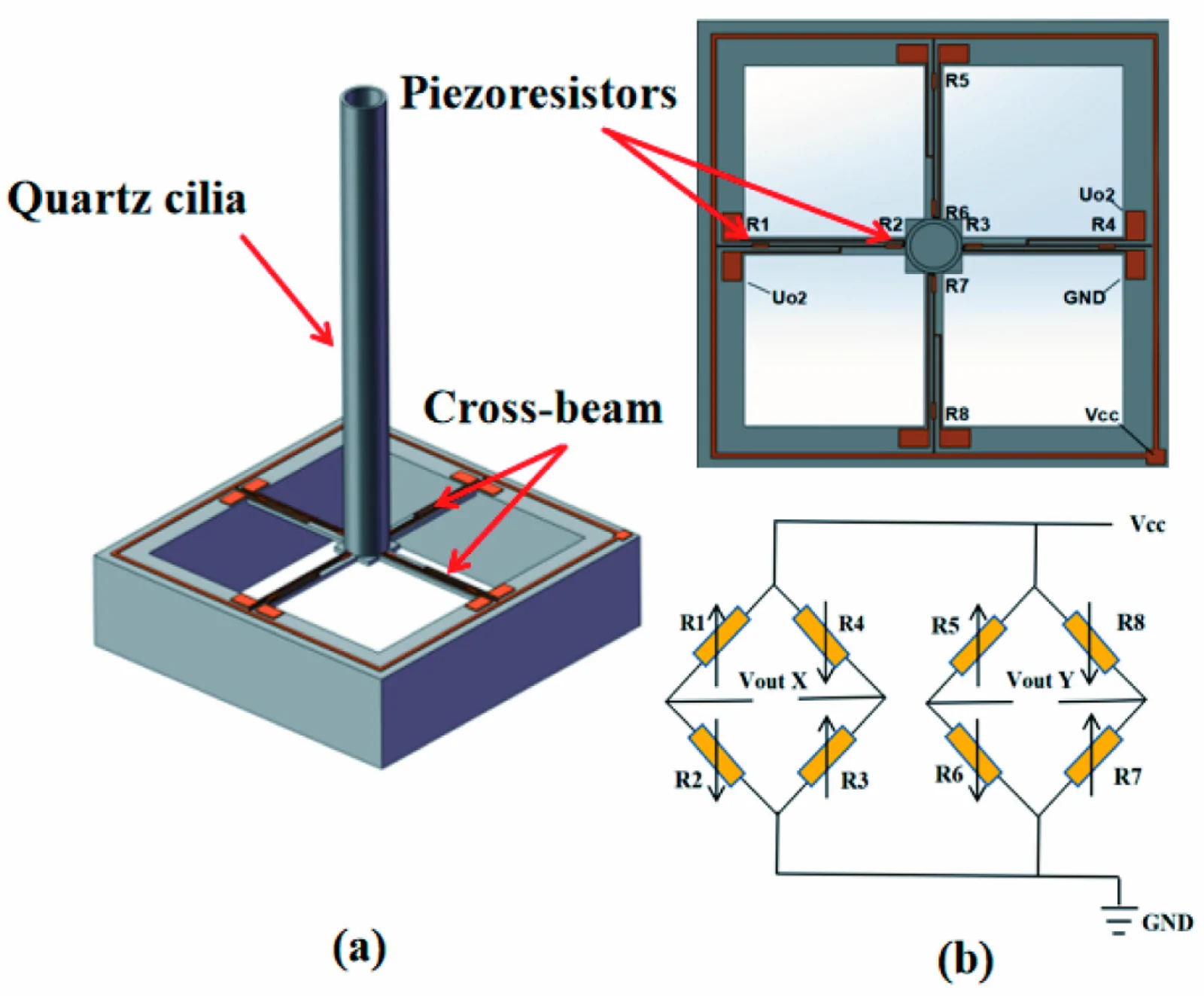
(**a**) Schematic diagram of the quartz cilium. (**b**) Wheatstone bridge.

Based on the sensing mechanism of the fish lateral line system, the sensitive structure of the quartz cilia vector hydrophone is designed using MEMS technology and the piezoresistive principle [18,19,20,21]. The sensitive structure of the vector hydrophone investigated in this paper consists of three components: a biomimetic cilium, a central proof mass, and a four-beam silicon microstructure, as illustrated in Figure 1a. The cilium is rigidly connected to four silicon cantilever beams arranged in a cross configuration via the central proof mass. When underwater acoustic waves act on the cilium, the acoustic pressure gradient drives the cilium into forced vibration. This vibration is transmitted through the central proof mass to the roots of the cantilever beams, inducing bending deformation in the beam structures. Piezoresistors, integrated via MEMS processes in the high-stress regions on the surfaces of the cantilever beams, exhibit resistance changes in response to stress variations. These changes are subsequently converted into a differential voltage signal through a Wheatstone bridge, thereby completing the acoustic-to-electrical transduction. Eight piezoresistors are distributed across the four cantilever beams, forming two Wheatstone bridges. The schematic diagram of the Wheatstone bridge is shown in Figure 1b. When the bridge is excited by a DC voltage of 5 V, the variations in the X-channel and Y-channel are output through $V_{\mathrm{outX}}$ and $V_{\mathrm{outY}}$, respectively.

### 2.2. Analysis of Stress and Natural Frequency

**Figure 2:**
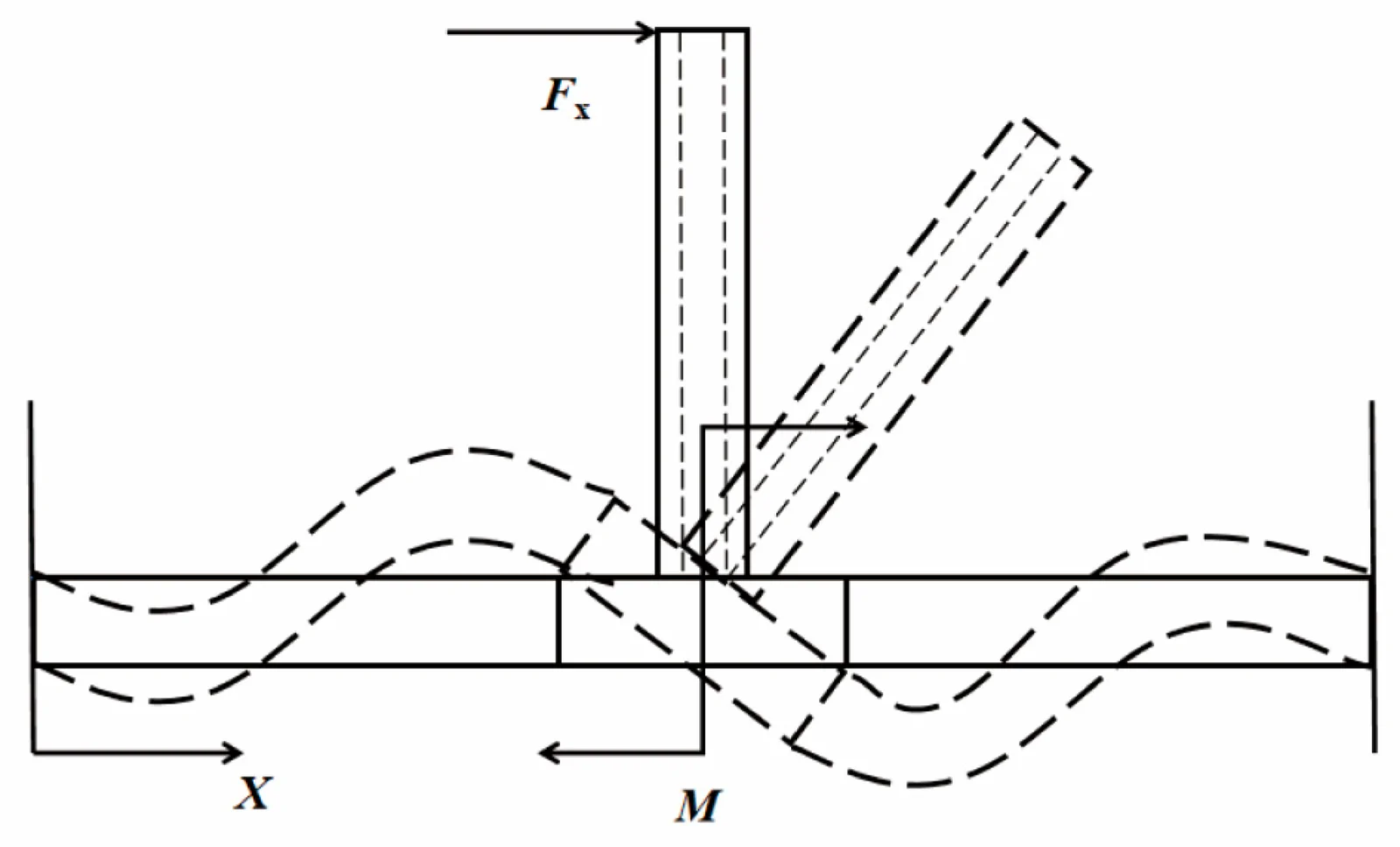
Force analysis diagram of the structure.

According to the mechanics of materials, the horizontal force $F_{\left(H\right)}$, as shown in Figure 2, is generated by the cilium under the action of the acoustic pressure is transmitted to the central proof mass and causes the cantilever beams to bend. For a single cantilever beam in the X-direction, the torsional effect transmitted through the central mass block by the cantilever beam in the y-direction is negligible. The stress σ(x) at an arbitrary point x along the beam can be expressed as:

(1) $$\sigma (x)=\pm \frac{L^{2}+3aL-3x(a+L)}{(4+\frac{6\beta }{1+\mu })L^{2}+12aL+12a^{2}}M\pm \frac{F_{H}}{bt}$$

where L, b, and t are the length, width, and thickness of the cantilever beam, respectively; a is the half-side length of the central proof mass; and M is the bending moment acting at the root of the beam.

Typical underwater targets, such as submarines and surface ships, have radiated noise whose energy centroid is predominantly concentrated in the frequency band of 100–1000 Hz [22]. Acoustic waves within this band exhibit long propagation distances and slow attenuation in water, making them the primary information carriers for underwater target detection and identification. Therefore, the operating bandwidth of a hydrophone must cover this core frequency range. The operating bandwidth of a hydrophone is closely related to the natural frequency of its sensitive structure, which essentially determines the upper-frequency limit to which the mechanical system can effectively respond.

The natural frequency of the sensitive structure can be expressed as:

(2) $$f=\frac{1}{2\pi }\sqrt{\frac{K}{m}}=\frac{1}{2\pi }\sqrt{2\frac{Ebt^{3}}{\rho \pi r^{2}lh^{3}}\left(\frac{a^{2}}{4l^{2}}+\frac{a}{2l}+\frac{1}{3}\right)}$$

where a is a quantity related to the transverse free vibration of the cilium, and K is the equivalent stiffness coefficient associated with the transverse free vibration of the cilium.

## 3. Simulation Analysis

### 3.1. Structural Design

Based on the mechanical analysis, this paper proposes a quartz hollow cilium-sensitive structure. The cilium is fabricated from transparent quartz capillary tubes, with the material parameters listed in Table 1. Compared with conventional resin materials, quartz has a higher stiffness that can effectively reduce mechanical energy dissipation during vibration, thereby improving the acousto-mechanical conversion efficiency. Meanwhile, its density is close to that of water, which facilitates co-vibration of the cilium with water particles. The cilium adopts a hollow cylindrical configuration, which, under the condition of identical outer diameter, significantly increases the acoustic receiving area and enhances the acoustic driving force. The hollow design also reduces the added mass, helping to maintain a high natural frequency to preserve the operating bandwidth.

The cantilever beams and the central proof mass are fabricated using mature silicon-based MEMS processes, The basic dimensions of the sensitive unit are presented in Table 2, which have been determined by taking into account both historical design experience and existing MEMS process constraints.

### 3.2. Parametric Simulation

**Figure 3:**
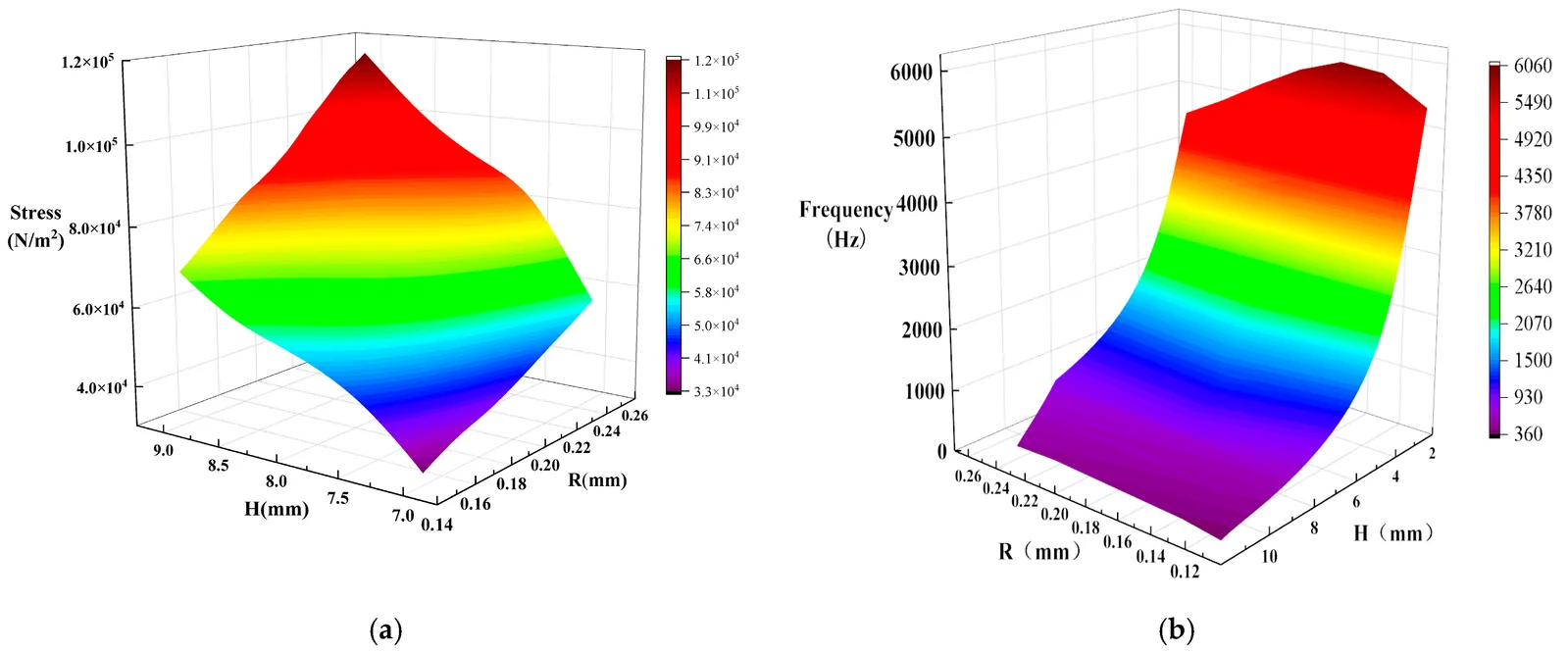
Relationship between cilium geometric dimensions and structural performance: (**a**) maximum stress versus cilium outer radius and height; (**b**) natural frequency versus cilium outer radius and height.

A three-dimensional finite element model was established using the COMSOL Multiphysics 6.3 platform, and a systematic optimization of the key geometric parameters of the quartz cilium was conducted. Taking the outer radius R and height H of the cilium as the primary design variables, their influence on the maximum stress on the beam and the natural frequency of the structure was analyzed. The simulation results are shown in Figure 3: the maximum stress on the beam increases monotonically with increasing R and H, while the natural frequency decreases monotonically with increasing R and H, confirming the trade-off relationship between sensitivity and bandwidth.

Considering both the detection requirement that underwater target radiated noise energy is predominantly concentrated in the 100–1000 Hz frequency band and the feasibility of MEMS fabrication, the optimized dimensions of the quartz cilium were finally determined, as shown in Table 3.

### 3.3. Stress Distribution and Modal Analysis

**Figure 4:**
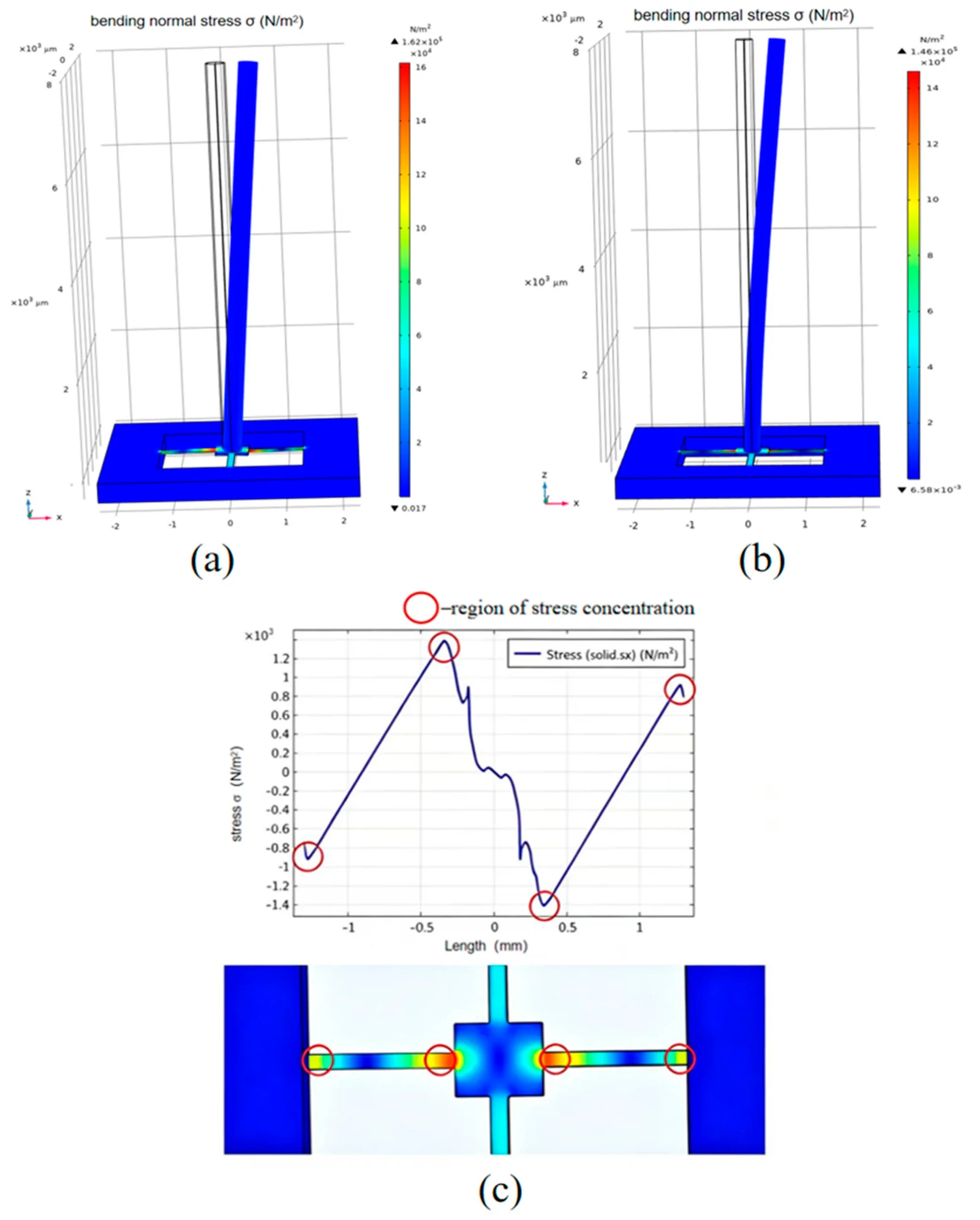
Stress distribution of the sensitive structure: (**a**) hollow cylindrical quartz structure surface stress distribution; (**b**) soild cylindrical quartz structure surface stress distribution; (**c**) stress distribution along the beam.

Based on the optimized dimensions, a simulated acoustic pressure excitation of 1 Pa was applied to the entire structure, and the resulting hollow cylindrical surface stress distribution of the sensitive structure is shown in Figure 4a. The maximum bending normal stress occurs at the roots of the four cantilever beams, with a value of 1.62 × 10^5^ N/m^2^. Compared with the conventional solid polymer cilium structure, which exhibits a maximum bending normal stress of 1.99 × 10^4^ N/m^2^ [11,12], the stress is enhanced by a factor of approximately 8.14. According to the sensitivity calculation formula, 20log_10_(8.14) ≈ 18.2 dB, which providing a preliminary estimate that the sensitivity can be improved by approximately 18.2 dB. The surface stress distribution of the obtained solid quartz sensitive structure is shown in Figure 4b. According to the sensitivity calculation formula, 20log_10_(1.36) ≈ 2.67 dB, the hollow quartz cilia structure achieves a sensitivity improvement of approximately 2.67 dB compared to the solid structure.

The bending normal stress distribution curve was extracted along the central axis of the upper surface of the cantilever beam in the X-direction, as shown in Figure 4c. The results show that the maximum stress regions are concentrated at the two ends of the beam-near the central proof mass side and near the fixed-end frame side. Accordingly, the optimal placement positions for the piezoresistors are determined to be the high-stress linear regions at the root portions at both ends of the beam.

**Figure 5:**
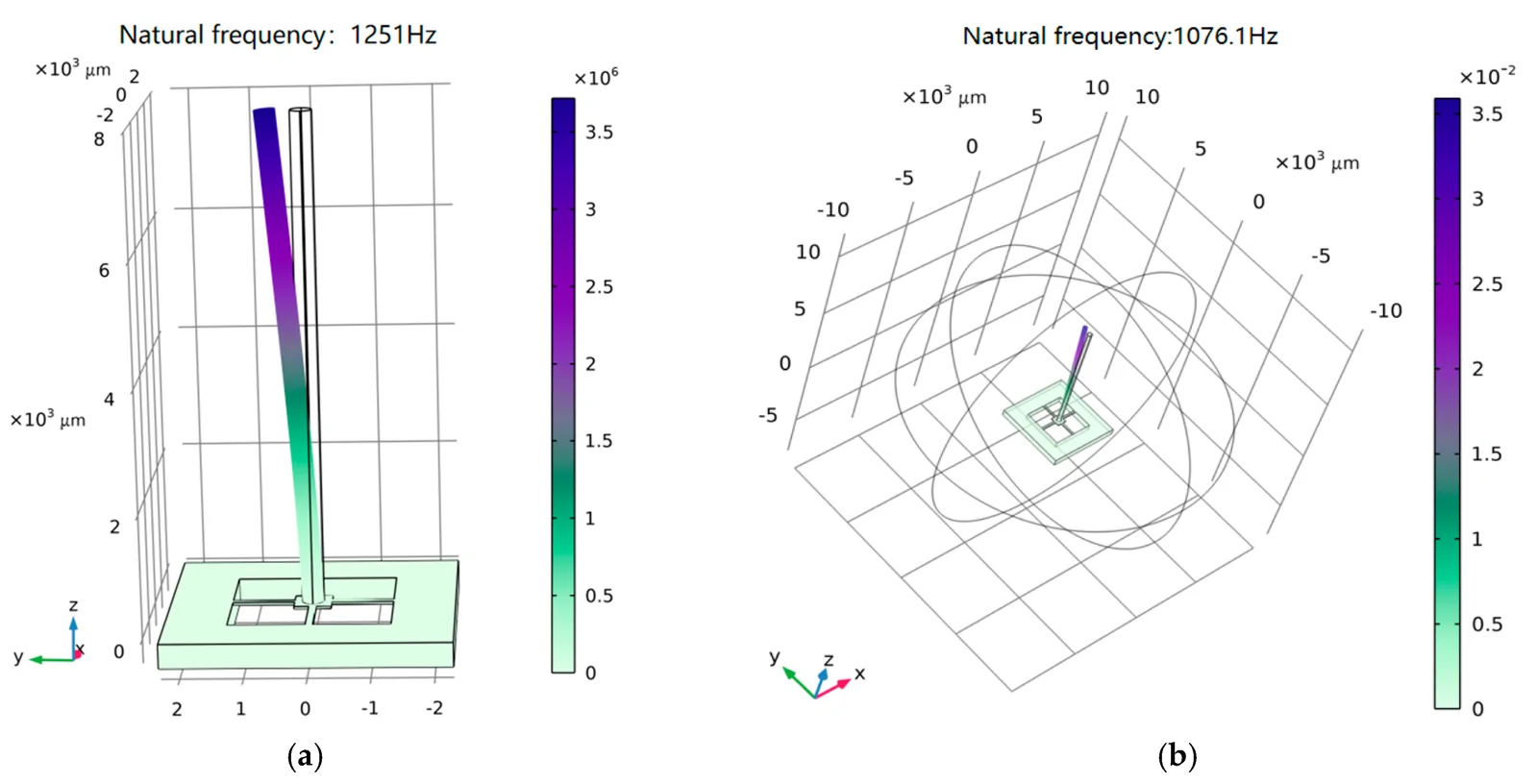
(**a**) Natural frequencies of the sensitive structure in air. (**b**) Natural frequency of the sensitive structure in water.

The cilium-based vector hydrophone operating in an underwater environment exhibits typical fluid-structure interaction behavior [23,24,25]. Modal analysis of the structure was conducted using COMSOL Multiphysics 6.3 software. The first-order vibration modes and corresponding natural frequencies of the structure in air and in water were obtained, as shown in Figure 5, which are 1251 Hz and 1076.1 Hz, respectively. The simulation results meet the bandwidth requirements of the hydrophone.

## 4. Manufacturing and Testing

### 4.1. Manufacturing

**Figure 6:**
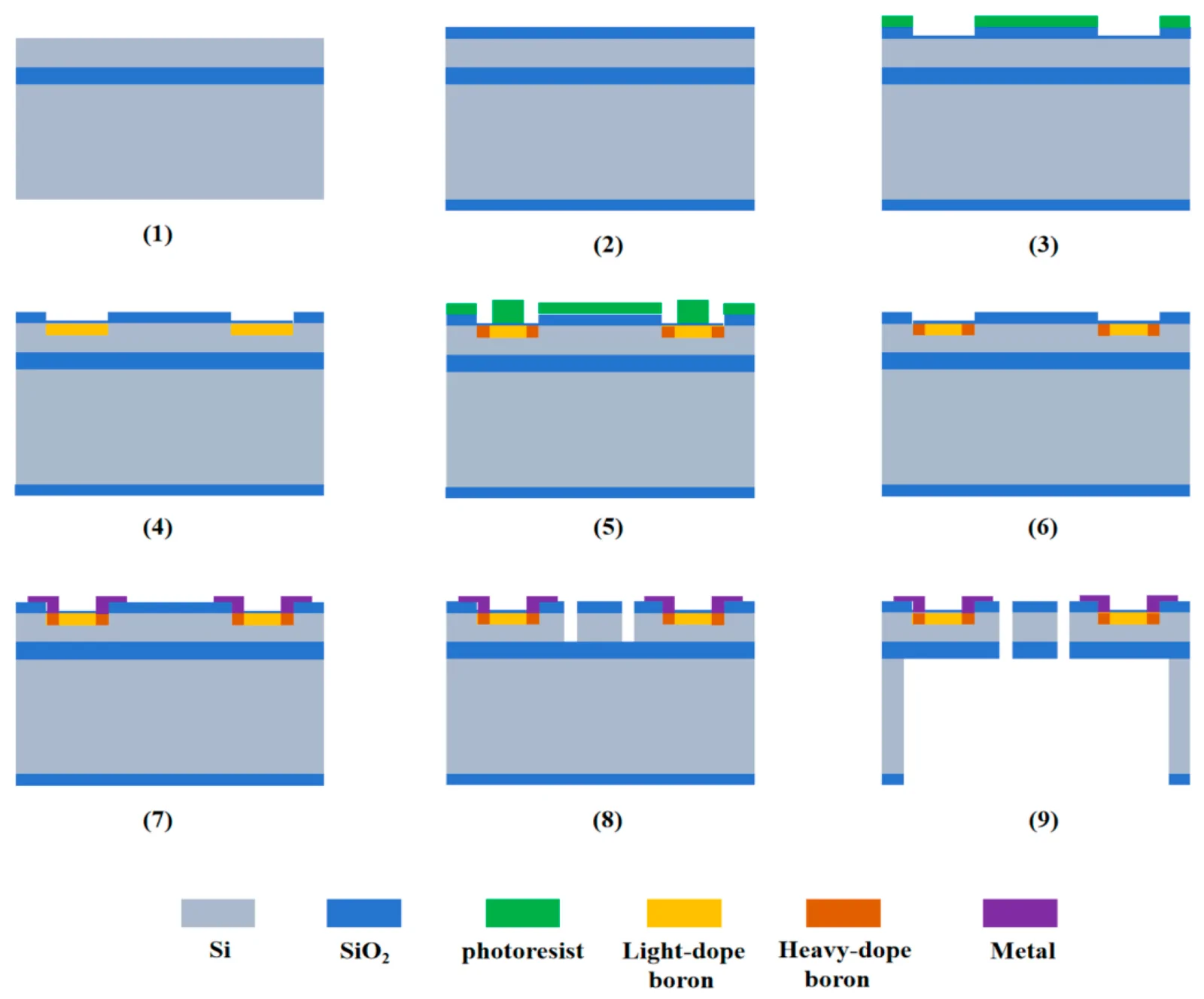
MEMS fabrication process flow of the vector hydrophone. (**1**) SOI handle wafer; (**2**) double-sided oxidation; (**3**) first lithography and SiO_2_ etching; (**4**) boron light implantation and photoresist removal; (**5**) second lithography and boron heavy implantation; (**6**) surface SiO_2_ removal, annealing, and photoresist removal; (**7**) sputtering, third lithography, etching, and annealing to form ohmic contact; (**8**) fourth lithography and front-side etching; (**9**) fifth lithography, back-side etching, and beam release.

**Figure 7:**
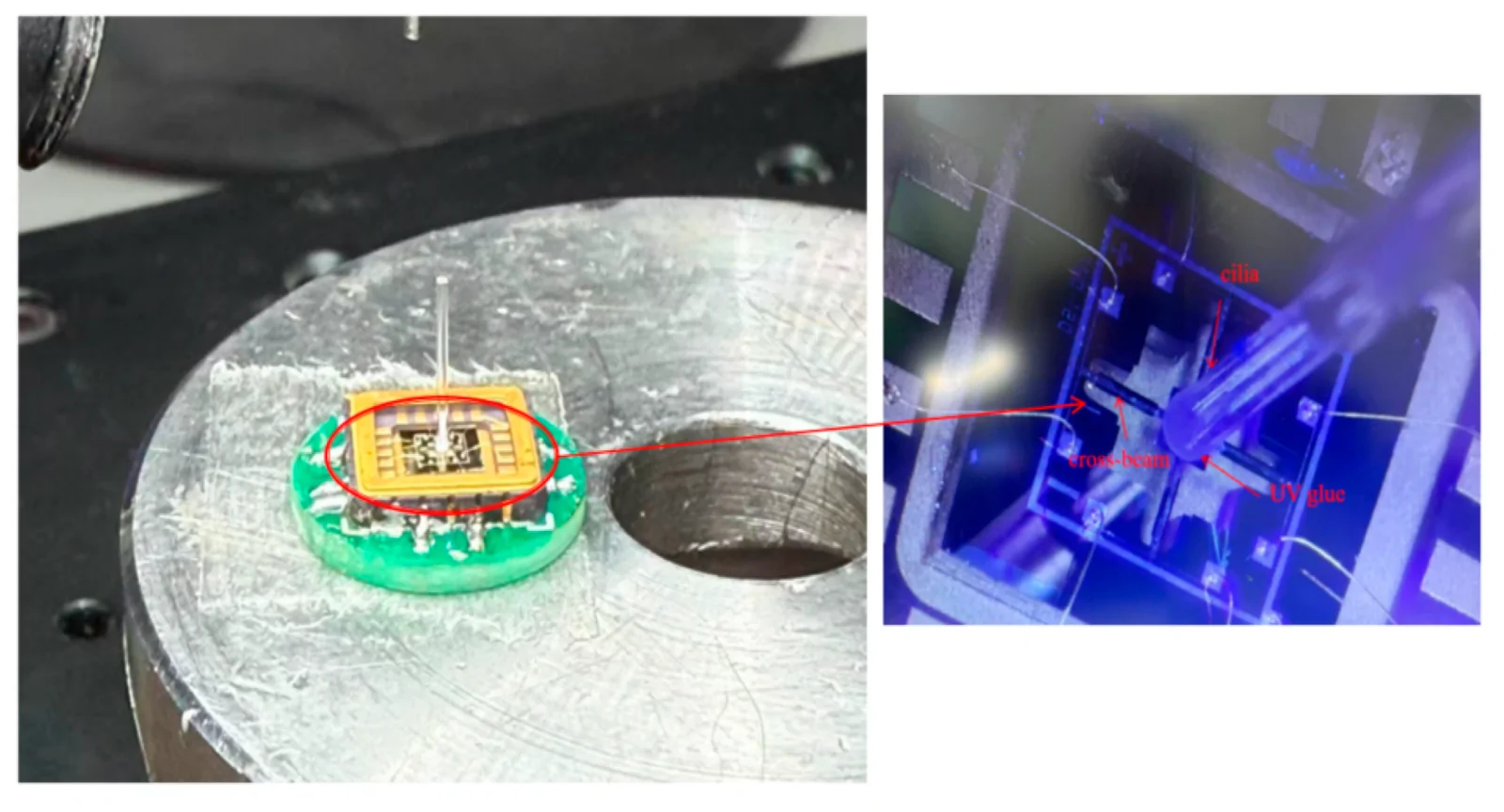
Photograph of the sensitive structure.

**Figure 8:**
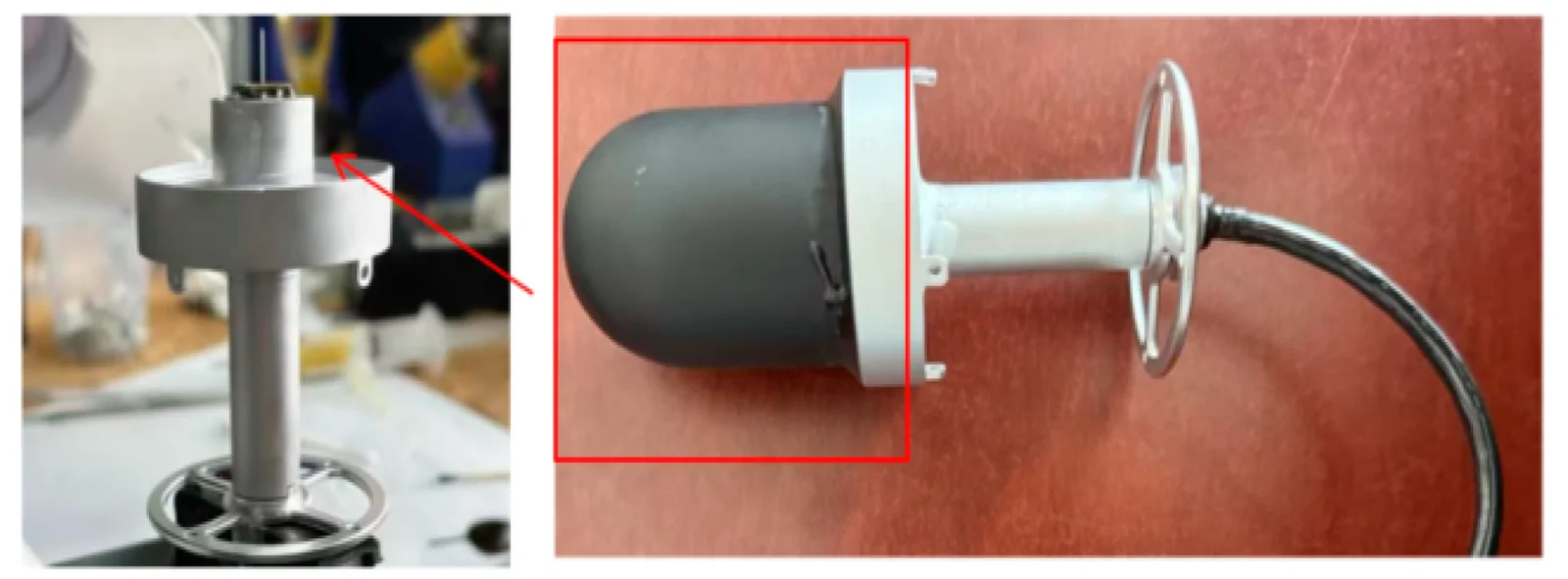
Photograph of the QCVH prototype.

Based on the geometric parameters determined through simulation optimization, a hollow quartz biomimetic cilium was custom-fabricated. The sensitive unit of the vector hydrophone was fabricated using MEMS technology through nine core microfabrication processes, including ion implantation, ohmic contact formation, front-side etching, and back-side release. The process steps are illustrated in Figure 6. The fabricated chip was mounted onto a custom Printed Circuit Board (PCB) and a ceramic package, and electrical interconnection was achieved via wire bonding to connect with the subsequent weak signal conditioning circuit. The quartz cilium was vertically bonded onto the central proof mass of the cross-beam structure using a specialized integration platform, using UV glue. The UV adhesive used is a low-modulus epoxy type, and the stress relaxation and material softening effects caused by viscoelasticity can be neglected under this frequency range and room temperature operating conditions, allowing the bonding interface to be considered a rigid connection. The sensitive structure is shown in Figure 7. Finally, the sensitive structure was encapsulated in a metal housing to form a complete prototype. The photograph of the QCVH (Quartz Cilium Vector Hydrophone) is presented in Figure 8.

### 4.2. Testing

**Figure 9:**
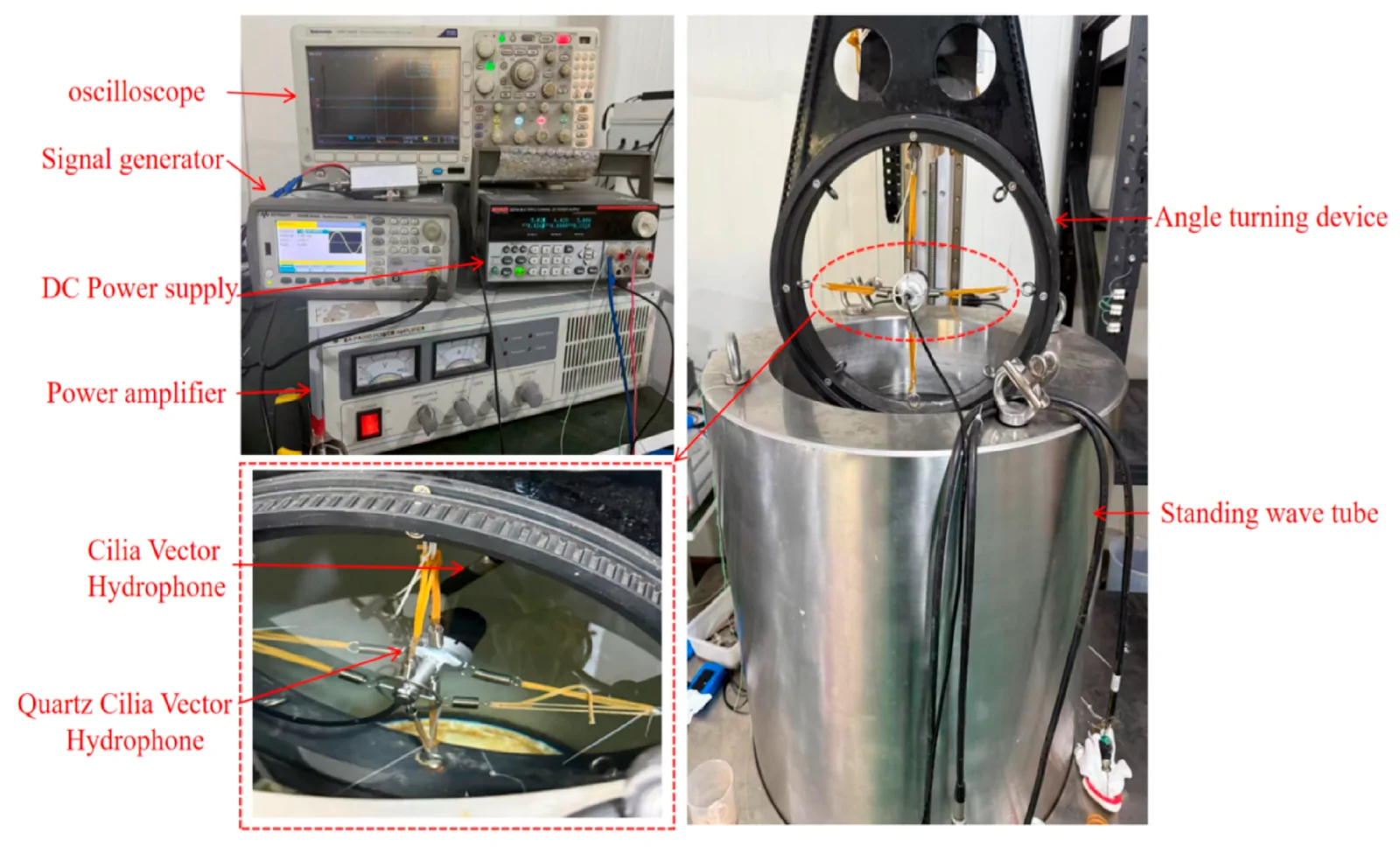
Hydrophone calibration system.

The hydrophone automatic calibration system was used to test the sensitivity and “figure-of-eight”-character directivity of the QCVH. The calibration system is shown in Figure 9. For receiving sensitivity measurements, the experimental test adopted for calibration is the standard comparison technique, which compares the output voltage of a hydrophone with that of the standard hydrophone [24]. There is a standing wave sound field in the standing wave barrel. According to the sound pressure distribution theory of the standing wave sound field, a 1/3 octave band of the sound signal is taken as the calibration frequency. The calculation formula for the sound pressure sensitivity of the measured vector hydrophone is shown in Equation (3):

(3) $$M_{x}=20\mathrm{lg}(\frac{e_{x}}{e_{0}}\frac{\sin kd}{\cos kd})+M_{0}$$

where $M_{x}$ is the sensitivity of the vector hydrophone under test, $M_{0}$ is the sensitivity of the standard vector hydrophone (the sensitivity of the standard hydrophone used in this experiment is −196.7 dB at 1 kHz, re 1 V/μPa), $e_{x}$ and $e_{0}$ represent the output voltage measured by the vector hydrophone under test and the standard hydrophone, respectively, k is the wavenumber at different frequencies, and d is the immersion depth of the vector hydrophone and the standard hydrophone in the standing wave tube.

**Figure 10:**
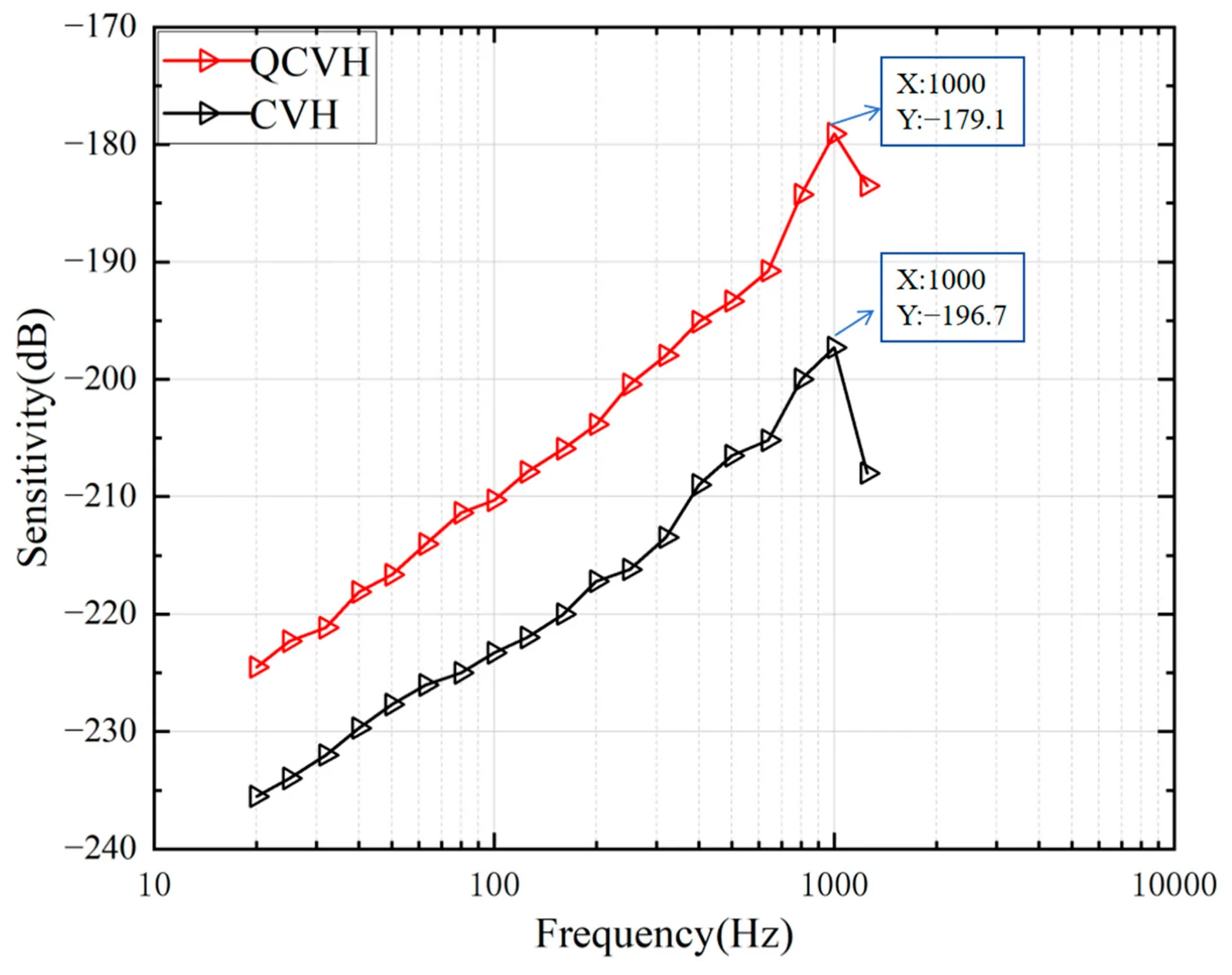
Sensitivity curves of the QCVH and the conventional CVH.

Figure 10 presents the sensitivity calibration curve of the QCVH. Compared with the sensitivity curve of the conventional cilium structure hydrophone (CVH), the effective operating bandwidth of the quartz cilia vector hydrophone is 20–1000 Hz, covering the main energy concentration band of underwater target radiated noise. At 1000 Hz, its sensitivity reaches −179.1 dB (1 kHz, re 1 V/μPa), which represents an improvement of approximately 17.6 dB over the conventional cilium structure hydrophone. This enhancement is in close agreement with the 18.2 dB predicted by the simulation in Section 3, thereby validating the accuracy and effectiveness of the simulation model and the design methodology. Furthermore, the sensitivity of an acoustic pressure gradient hydrophone follows a frequency-dependent relationship of an increase of approximately 6 dB per octave. The measured curve of the quartz cilia vector hydrophone exhibits an upward trend consistent with this characteristic within the 20–1000 Hz range, demonstrating its favorable gradient sensing properties and confirming that its operating principle aligns with the design expectations. To comprehensively evaluate the overall performance of QCVH, Table 4 summarizes the comparison results of it with various improved type ciliary MEMS vector hydrophones reported in recent years in key indicators. By comparing the table, it can be seen that QCVH has a significant advantage in the sensitivity-bandwidth comprehensive performance in the low-frequency range.

The directivity of a hydrophone refers to its spatial response characteristic, namely the variation of its sensitivity with the incident direction of the acoustic wave. It is a parameter that quantifies the hydrophone’s ability to receive acoustic signals from different directions, and it determines the hydrophone’s capability to spatially discriminate the position of sound sources and suppress interfering noise.

The directivity formula of the vector hydrophone is as follows:

(4) $$L=20\mathrm{lg}(\frac{e_{\theta }}{e_{\max}})$$

$e_{\theta }$ and $e_{\max}$ represent the output voltage of the vector hydrophone under test at an arbitrary angle and the output voltage in the direction of the sensitive axis, respectively.

**Figure 11:**
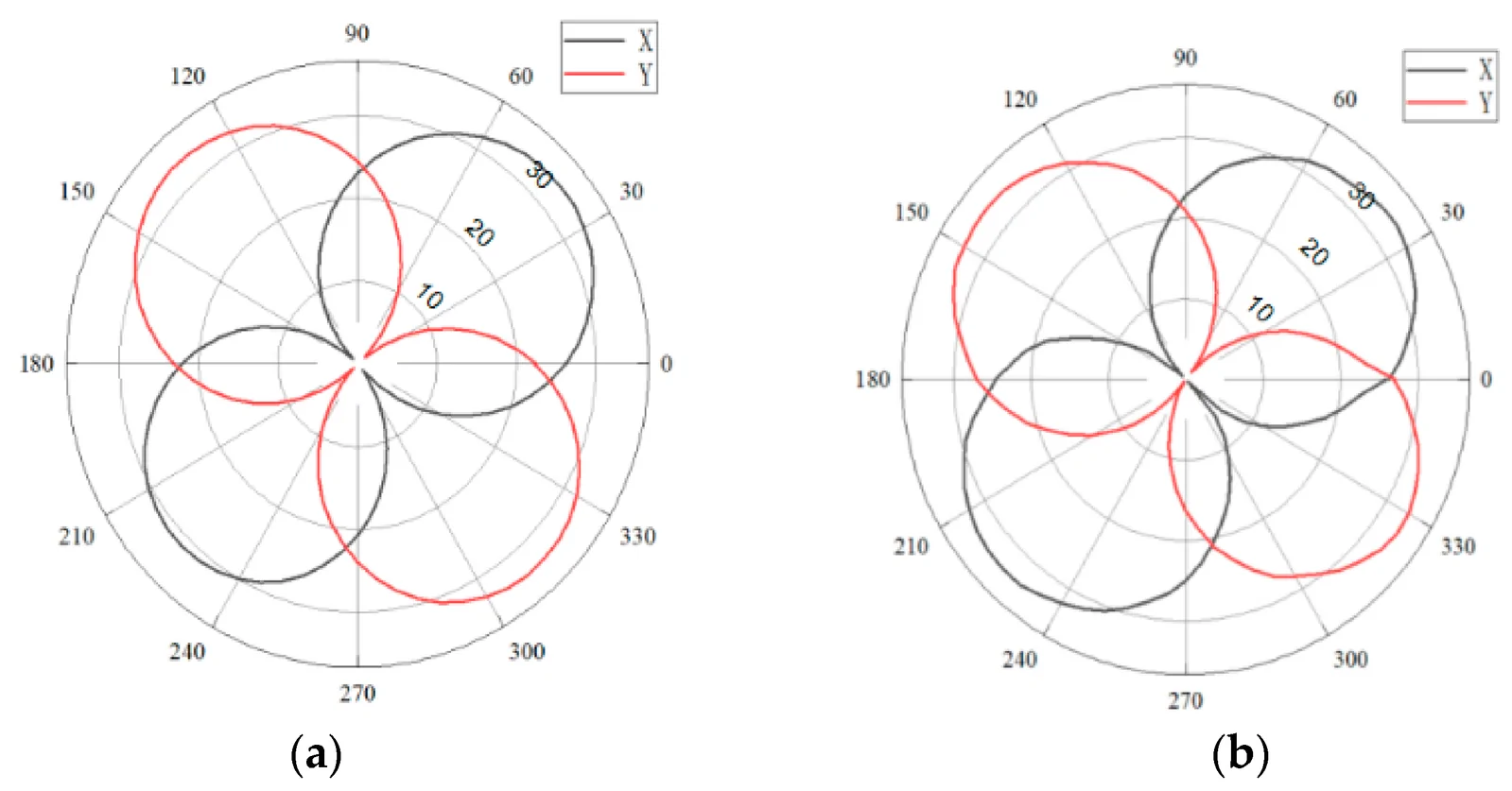
Directivity patterns of QCVH. (**a**) 315 Hz, (**b**) 630 Hz.

Based on the above formula, the measured data were processed to obtain the “figure-of-eight” directivity patterns of the hydrophone at two characteristic frequencies, 315 Hz and 630 Hz, as shown in Figure 11.

The directivity test results show that the quartz cilium vector hydrophone achieves null depths of 33 dB at 315 Hz and 32 dB at 630 Hz. The null depths at both frequencies exceed 30 dB, indicating that the hydrophone possesses excellent localization capability and can effectively suppress interference signals from non-sensitive-axis directions. As the frequency increases, the directivity pattern remains stable, with only a slight decrease in null depth, demonstrating that the sensor maintains good directional performance over a relatively wide frequency range. The directivity test results not only validate the spatial resolution capability of the device but also indirectly reflect the assembly precision of the cilium and the cross-beam structure. The ideal figure-8 directivity pattern and its symmetry indicate that the cilium perpendicularity, the geometric symmetry of the cross-beam, and the matching of the piezoresistors all meet the design requirements, thereby establishing a quality foundation for subsequent engineering applications.

## 5. Conclusions

This paper proposes a MEMS vector hydrophone based on a hollow quartz cilium. Through synergistic optimization of intrinsic material properties and structural geometry, the proposed design effectively alleviates the inherent trade-off between sensitivity and operating bandwidth that exists in conventional cilium-type hydrophones, achieving simultaneous improvements in both low-frequency sensitivity and operational bandwidth. Systematic parametric simulations and dry/wet modal analyses were conducted using the COMSOL Multiphysics 6.3 platform, which verified that the hollow quartz cilium design not only enhances the stress level on the cantilever beams but also maintains the first-order natural frequency in water at 1076.1 Hz, ensuring that the operating frequency band covers 20–1000 Hz. Finally, experimental results demonstrate that the fabricated hydrophone attains a sensitivity of –179.1 dB (re 1 V/μPa) at 1000 Hz, representing an improvement of approximately 17.6 dB over the conventional polymer cilium structure. The frequency response curve exhibits excellent agreement with the theoretical 6 dB per octave increase characteristic of pressure-gradient sensors across the 20–1000 Hz band. Meanwhile, the device exhibits typical “figure-of-eight” dipole directivity patterns at both 315 Hz and 630 Hz, with null depths of 33 dB and 32 dB, respectively, confirming its superior vector directional capability. In summary, the proposed quartz cilium vector hydrophone meets the essential requirements for low-frequency detection of ship-radiated noise and provides a viable technical pathway and experimental basis for material innovation and structural optimization of high-performance MEMS vector hydrophones.

## Figures and Tables

**Table 1:** Parameters of structure.

Material	Density (g/cm^3^)	Elastic Modulus (GPa)	Poisson Coefficient
Quartz	2.2	76.7	0.17
Si	2.33	160	0.27

**Table 2:** Dimensions of the hydrophone sensitive unit.

Structure Name	Parameter (μm)
l (Length of beam)	1000
b (Width of beam)	120
d (Thickness of beam)	40
a (Half-length of the central proof mass)	600

**Table 3:** Dimensions of the quartz cilium.

Structure Name	Parameter (mm)
H (height of cilium)	8
$r_{1}$ (outer radius of cilium)	0.15
$r_{2}$ (inner radiuss of cilium)	0.1

**Table 4:** Performance comparison of hydrophones.

Reference	Sensor	Type	Bandwidth	Sensitivity
Liu, Y. et al.	“Lollipop-shaped” hydrophone [26]	piezoresistive	20–500 Hz	−183 dB@500Hz
Geng, Y. et al.	Hollow Mushroom-Like Cilia MEMS Vector Hydrophone [15]	piezoresistive	20–1000 Hz	−180.9 dB@1000Hz
Zhu, S. et al.	cap-shaped cilia MEMS vector hydrophone [17]	piezoresistive	20–1000 Hz	−182.7 dB@1000Hz
Ren, W. et al.	Crossed-circle cilia MEMS vector hydrophone [14]	piezoresistive	20–1250 Hz	−184.5 dB@1250Hz
This work	Quartz Cilia MEMS Vector Hydrophone	piezoresistive	20–1000 Hz	−179.1 dB@1000Hz

## Data Availability

Data available on request from the authors.
